# Inhibition of GSK3 Phosphorylation of β-Catenin via Phosphorylated PPPSPXS Motifs of Wnt Coreceptor LRP6

**DOI:** 10.1371/journal.pone.0004926

**Published:** 2009-03-18

**Authors:** Geng Wu, He Huang, Jose Garcia Abreu, Xi He

**Affiliations:** 1 F. M. Kirby Neurobiology Center, Children's Hospital Boston, Harvard Medical School, Boston, Massachusetts, United States of America; 2 Instituto de Ciencias Biomedicas, Universidade Federal do Rio de Janeiro, Rio de Janeiro, Brazil; University of Hong Kong, Hong Kong

## Abstract

The Wnt/β-catenin signaling pathway plays essential roles in cell proliferation and differentiation, and deregulated β-catenin protein levels lead to many types of human cancers. On activation by Wnt, the Wnt co-receptor LDL receptor related protein 6 (LRP6) is phosphorylated at multiple conserved intracellular PPPSPXS motifs by glycogen synthase kinase 3 (GSK3) and casein kinase 1 (CK1), resulting in recruitment of the scaffolding protein Axin to LRP6. As a result, β-catenin phosphorylation by GSK3 is inhibited and β-catenin protein is stabilized. However, how LRP6 phosphorylation and the ensuing LRP6-Axin interaction lead to the inhibition of β-catenin phosphorylation by GSK3 is not fully understood. In this study, we reconstituted Axin-dependent β-catenin phosphorylation by GSK3 and CK1 in vitro using recombinant proteins, and found that the phosphorylated PPPSPXS peptides directly inhibit β-catenin phosphorylation by GSK3 in a sequence and phosphorylation-dependent manner. This inhibitory effect of phosphorylated PPPSPXS motifs is direct and specific for GSK3 phosphorylation of β-catenin at Ser33/Ser37/Thr41 but not for CK1 phosphorylation of β-catenin at Ser45, and is independent of Axin function. We also show that a phosphorylated PPPSPXS peptide is able to activate Wnt/β-catenin signaling and to induce axis duplication in Xenopus embryos, presumably by inhibition of GSK3 in vivo. Based on these observations, we propose a working model that Axin recruitment to the phosphorylated LRP6 places GSK3 in the vicinity of multiple phosphorylated PPPSPXS motifs, which directly inhibit GSK3 phosphorylation of β-catenin. This model provides a possible mechanism to account, in part, for inhibition of β-catenin phosphorylation by Wnt-activated LRP6.

## Introduction

The Wnt/β-catenin signal transduction pathway plays central roles in many aspects of cell proliferation and differentiation, such as segment polarity determination in *Drosophila*, dorsal-ventral axis formation in *Xenopus*, and homeostasis of the mammalian gastrointestinal tract [1]–[3]. The onco-protein β-catenin is a central component of the Wnt signaling pathway. Its protein level inside the cell is tightly regulated by phosphorylation-dependent and ubiquitin-mediated degradation, and deregulated β-catenin protein level leads to many types of human cancers, such as colorectal carcinoma and melanoma [4]. In the absence of secreted Wnt ligands, cytosolic β-catenin is phosphorylated at Ser45 by the priming kinase casein kinase 1 (CK1). Consequently, glycogen synthase kinase 3 (GSK3), in complex with Axin and *adenomatous polyposis coli* (APC), phosphorylates β-catenin at Thr41, Ser37, and Ser33 [5]–[15]. Ser33 and Ser37 doubly-phosphorylated β-catenin is specifically recognized by β-TrCP [16]–[22], a subunit of the SCF^β-TrCP^ E3 ubiquitin ligase complex. The SCF^β-TrCP^ ubiquitin ligase poly-ubiquitinates β-catenin, leading to β-catenin degradation via the proteosome pathway [23], [24]. In the presence of Wnt ligands, the activation of the Wnt pathway results in inhibition of β-catenin phosphorylation at Ser33 and Ser37 (and Thr41) by GSK3, thereby preventing β-catenin ubiquitination and degradation. Stabilized β-catenin translocates into the nucleus and complexes with members of the T cell factor (TCF)/lymphoid enhancer factor (LEF) family of transcription factors [25]–[27], leading to the activation of Wnt/β-catenin responsive genes such as c-myc and cyclin D1 [28], [29]. Therefore, inhibition of amino-terminal phosphorylation of β-catenin by GSK3 is a central step in Wnt/β-catenin signaling.

Wnt activates the β-catenin pathway via two distinct classes of receptors on the cell surface: one is a member of the Frizzled family of seven-transmembrane receptors, and the other is a single transmembrane receptor referred to as LDL receptor related protein 6 (LRP6), or its relative LRP5. Wnt may induce a Frizzled-LRP6 coreceptor complex [30]–[33], which in turn triggers the phosphorylation of LRP6 intracellular domain at five conserved PPP(S/T)PX(S/T) motifs (referred to as PPPSPXS for simplicity) [34], [35]. The phosphorylated PPPSPXS motif provides an optimal binding site for Axin [34], [35], thereby recruiting Axin and likely associated proteins to the Frizzled-LRP6 receptor complex [33], [36] and leading to the inhibition of β-catenin phosphorylation. Importantly the phosphorylated PPPSPXS motif represents a key and minimal functional module of the Wnt receptor complex, since it is sufficient to trigger β-catenin signaling when transferred to a heterologous receptor [34], [35], [37]. PPPSPXS phosphorylation is carried out sequentially by GSK3 and CK1 [35], [37], [38] and is under the control by Frizzled and its downstream partner Dishevelled protein [39], [40].

How PPPSPXS phosphorylation and its recruitment of Axin result in inhibition of β-catenin phosphorylation remains a critical question. To address this issue we established an in vitro β-catenin phosphorylation system using recombinant Axin, GSK3 and CK1. We found that each of the multiple phosphorylated PPPSPXS peptides inhibits the phosphorylation of β-catenin at Ser33/Ser37/Thr41 by GSK3 in a sequence and phosphorylation-dependent manner. This inhibition is specific for GSK3, as these phospho-peptides do not affect β-catenin Ser45 phosphorylation by CK1, and occurs regardless of the presence or absence of Axin. We also found that a phosphorylated PPPSPXS peptide is able to activate Wnt/β-catenin signaling and to induce axis duplication in Xenopus embryos, presumably via inhibition of GSK3 in vivo. These results suggest a potential mechanism to account, in part, for the inhibition GSK3 phosphorylation of β-catenin by the activated LRP6. While this manuscript was in previous review processes, Cselenyi *et al.* reported that the LRP6 intracellular domain directly inhibits GSK3 phosphorylation of β-catenin in a PPPSPXS-dependent manner [41]. Our results based on studying individual phospho-PPPSPXS peptides are consistent with their main conclusion. However, while Cselenyi *et al.* suggested that LRP6 specifically inhibits GSK3 phosphorylation of β-catenin but not of other substrates [41], our data suggest that the phosphorylated PPPSPXS peptide behaves as a general GSK3 inhibitor.

## Results

### Reconstitution of Axin-dependent β-catenin amino-terminal phosphorylation by CK1 and GSK3 in vitro

**Figure 1:**
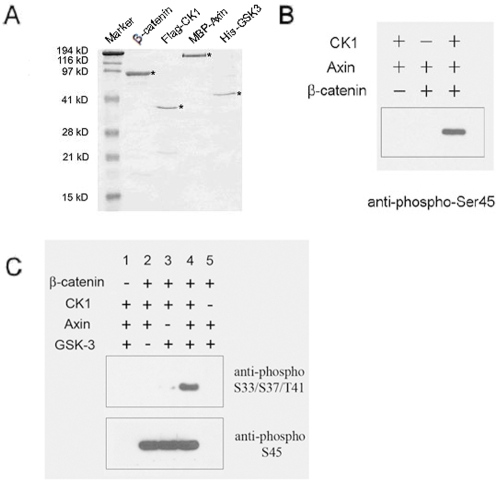
In vitro reconstitution of Axin-dependent and CK1 priming-dependent β-catenin phosphorylation by GSK3. *A*. Recombinant GST-β-catenin, Flag-CK1, MBP-Axin, and His-GSK3 proteins were expressed in bacteria or insect cells and purified by glutathione agarose, anti-Flag M2 agarose, amylose resin, or Ni-NTA resin, respectively. In the case of β-catenin, GST was cleaved via thrombin and purified away from β-catenin. * indicates each recombinant protein. *B*. β-catenin phosphorylation by CK1 was reconstituted in vitro using purified proteins. The phosphorylation reaction products were analyzed by western blotting using an anti-phospho-Ser45 β-catenin antibody. *C*. Axin-dependent phosphorylation by GSK3 was reconstituted in vitro using purified proteins. For Axin-dependent β-catenin phosphorylation in this and other figures, 0.43 µM of GSK3, 0.54 µM of CK1α, 0.21 µM of Axin, and 0.73 µM of β-catenin were used in each assay. The phosphorylation reaction products were analyzed by western blotting using an anti-phospho-Ser45 β-catenin antibody and an anti-phospho-Ser33/Ser37/Thr41 β-catenin antibody.

To study how β-catenin phosphorylation is regulated by upstream components of the Wnt pathway, we reconstituted an in vitro kinase assay for β-catenin amino-terminal phosphorylation using purified proteins. We overexpressed recombinant β-catenin, Axin, CK1β, and GSK3β proteins in either *E. coli* or baculovirus-infected insect cells, and purified these proteins to over 90% homogeneity by affinity chromatography (Figure 1A). We incubated purified β-catenin with Axin, CK1, and GSK3 protein in the presence of ATP and MgCl_2_ at 37°C for 3 hours. β-catenin phopshorylation was analyzed by immunoblotting using an antibody specific for Ser45-phosphorylation (by CK1) or an antibody specific for Ser33/Ser37/Thr41-phosphorylation (by GSK3).

When CK1 was present in the kinase reaction, β-catenin was strongly phosphorylated at Ser45 (Figure 1B). When CK1, Axin and GSK3 were all present in the kinase reaction, β-catenin was potently phosphorylated at Ser33/Ser37/Thr41 (Figure 1C, lane 4). Phosphorylation of β-catenin at Ser33/Ser37/Thr41 fully depended on GSK3 (Figure 1C, lane 2), and also required the presence of Axin and the priming phosphorylation by CK1 (Figure 1C, lanes 3 and 5). These results are consistent with an earlier report [42], and importantly, recapitulate the in vivo requirement of β-catenin phosphorylation. Therefore we have reconstituted an in vitro kinase assay for Axin-dependent β-catenin amino-terminal phosphorylation by CK1 and GSK3 using purified proteins.

### Design of phosphorylated PPPSPXS motif peptides

**Figure 2:**
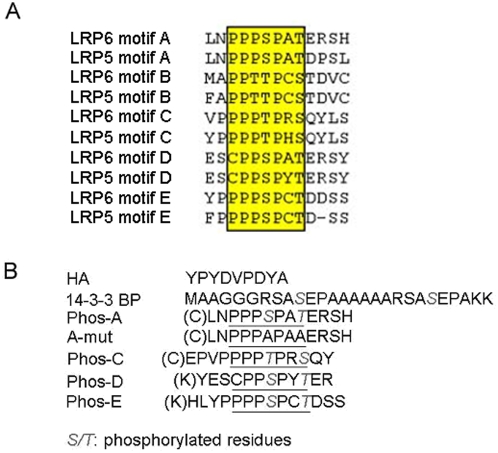
Peptide design according to the PPPSPXS motifs in human LRP6. *A*. Sequence alignment of the five PPPSPXS motifs in human LRP6 and LRP5 by the Cluster V program. The PPPSPXS motifs are highlighted in color and boxed. *B*. The sequences of synthetic peptides are shown. The PPPSPXS motifs in the peptides are underlined, and phosphorylated Ser/Thr residues are shown in italics. The C or K residue in the parenthesis at the amino terminus of peptides A, C, D, and E was introduced for protein conjugation purposes (during immunization for antibody production) [34], [37].

A single phosphorylated PPPSPXS motif is sufficient to activate β-catenin signaling in vivo [34], [37]. In order to investigate whether these PPPSPXS motifs might regulate β-catenin amino-terminal phosphorylation in our in vitro assay, we employed 4 phosphorylated PPPSPXS peptides corresponding to A, C, D, and E motifs of LRP6 (Figure 2A), referred to as Phos-A, Phos-C, Phos-D, and Phos-E, respectively, in which the two Ser/Thr residues in the PPPSPXS motifs were phosphorylated (Figure 2B). Although motif B behaves similarly to the other PPPSPXS motifs when tested in isolation in mammalian cells, motif B appears to be the least critical one in the wild type LRP6 [37], [43]. We therefore did not synthesize and test motif B. As controls, we also synthesized an HA peptide, a dually phosphorylated 14-3-3 binding peptide (14-3-3BP), and a mutant motif A peptide (A-mut), which harbors alanine replacement of the two phosphorylated Ser/Thr residues (Figure 2B) and which has been shown to be completely inactive in Wnt/β-catenin signaling in vivo [35].

### Phosphorylated PPPSPXS peptides inhibit β-catenin Ser33/Ser37/Thr41 phosphorylation by GSK-3

**Figure 3:**
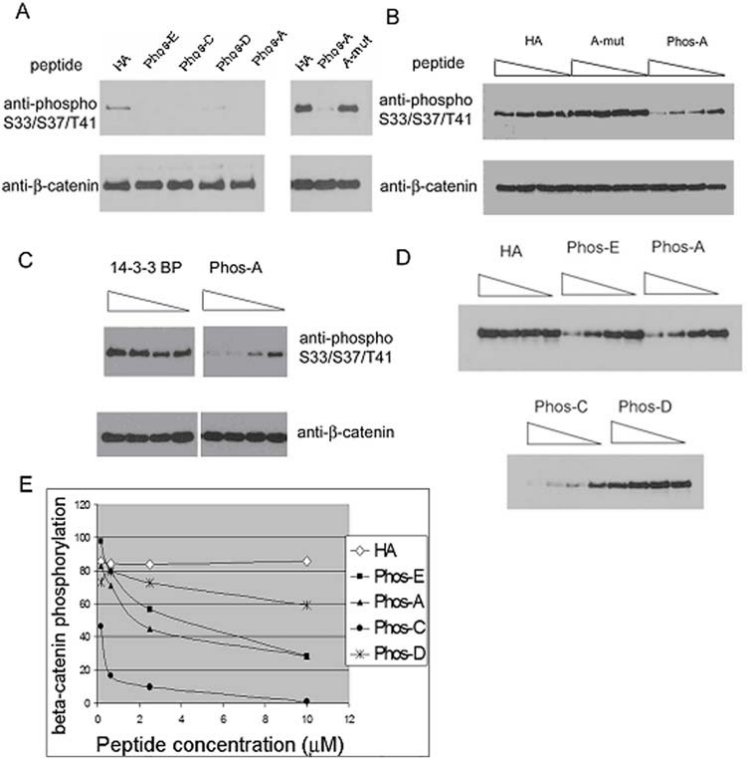
Phosphorylated PPPSPXS peptides inhibit β-catenin phosphorylation by GSK3 in vitro. *A*. The HA, Phos-E, Phos-C, Phos-D and Phos-A peptides (left panel) and the HA, Phos-A, and A-mut peptides (right panel) were included in the β-catenin phosphorylation assay. Each peptide was at 10 µM final concentration. *B*. Four-fold serial dilutions of HA, Phos-A, and A-mut peptides were included in the β-catenin phosphorylation assay. *C*. Four-fold serial dilutions of Phos-A, and 14-3-3BP peptides were included in the β-catenin phosphorylation assay. *D*. Four-fold serial dilutions of HA, Phos-E, Phos-A, Phos-C, and Phos-D peptides were included in the β-catenin phosphorylation assay. *E*. The result from D was quantified via Adobe Photoshop. β-catenin phosphorylation assays were performed in the presence of Axin and CK1 as in Figure 1C. Each peptide was at 10 µM, 2.5 µM, 0.63 µM, and 0.16 µM (four-fold serial dilutions) final concentration. The phosphorylation reaction products were analyzed by western blotting using an anti-phospho-Ser33/Ser37/Thr41 β-catenin antibody and an anti-β-catenin antibody.

When the phosphorylated PPPSPXS peptides were added to the in vitro β-catenin phosphorylation assay, the Phos-A, Phos-C, as well as Phos-E peptide each inhibited, interestingly, β-catenin phosphorylation at Ser33/Ser37/Thr41 by GSK3 (Figure 3A, left panel). The Phos-D peptide, which is atypical and has a CPPSPXS motif (Figure 2B), inhibited β-catenin phosphorylation by GSK3 to a less degree (Figure 3A, left panel). We note that in cultured cells motif D also exhibits significant less activity than A, C and E motifs [37]. The A-mut peptide failed to inhibit β-catenin phosphorylation (Figure 3A, right panel). As additional controls, neither the HA peptide nor the unrelated and dually phosphorylated peptide, 14-3-3BP, affected β-catenin phosphorylation by GSK3 (Figure 3B and 3C). Furthermore, when the amount of peptides used in the phosphorylation assay was titrated, Phos-A, Phos-C and Phos-E peptides inhibited β-catenin phosphorylation in a dose-dependent manner (Figure 3B, 3D, 3E), whereas Phos-D was significantly less active (Figure 3D, 3E). The HA and A-mut peptides did not inhibit β-catenin phosphorylation at all concentrations tested (Figure 3B, 3D, 3E). We note that the molar concentrations of phospho-PPPSPXS peptides employed in these titration assays were 0.4×, 1.5×, 6×, and 24× of that of GSK3 (see Methods).

### The phosphorylated PPPSPXS motif inhibits β-catenin phosphorylation by GSK3 but not by CK1 via an Axin-independent manner

**Figure 4:**
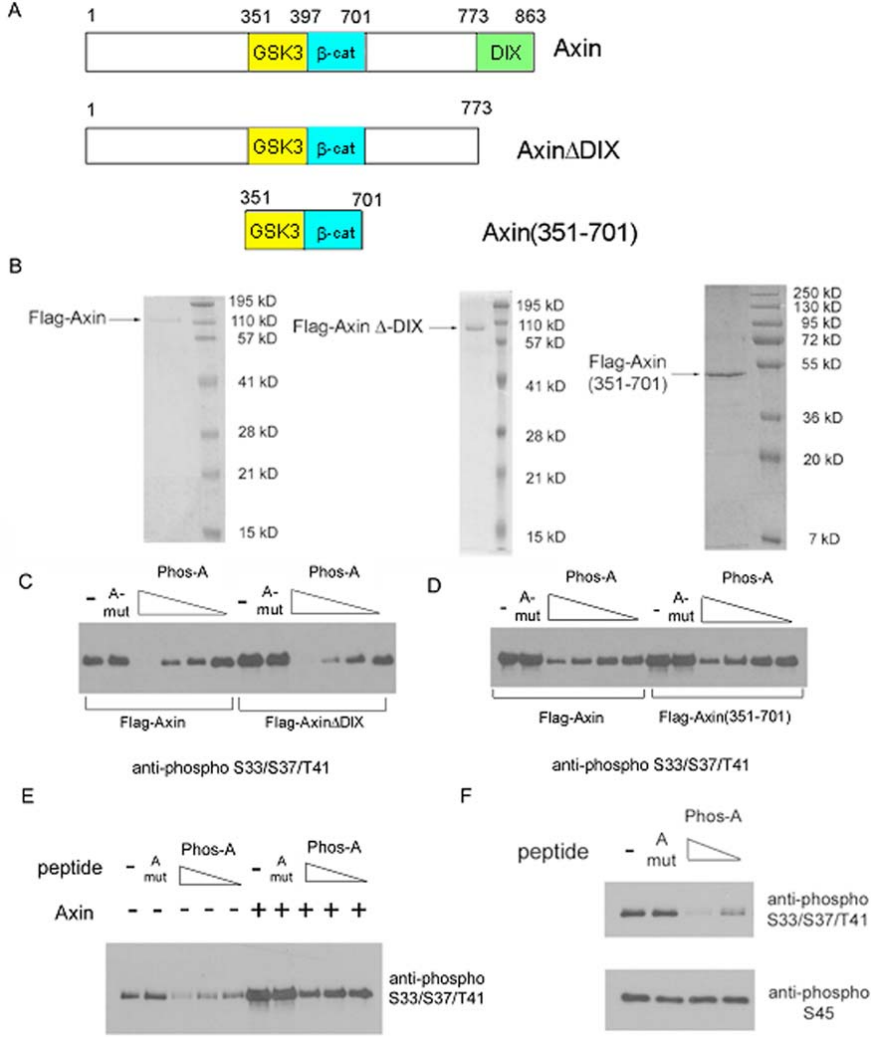
The inhibition of β-catenin phosphorylation by phosphorylated PPPSPXS peptides is specific for GSK3 and independent of Axin function. *A*. Different Axin constructs used in this study, the full length Axin (amino acid 1–863), AxinΔDix (1-773), and Axin(351-701) are shown. *B*. Purification of the full length Axin, AxinΔDix, and Axin(351-701) proteins. These Flagged tagged Axin and Axin fragments were expressed in HEK293T cells, purified via M2 agarose (Sigma) resin, and eluted by 0.2 mg/ml Flag peptides. *C* and *D*. The Phos-A peptide inhibited GSK3 phosphorylation of β-catenin in the presence of the full length Axin, or AxinΔDIX (C), or Axin(351-701) (D). Four-fold serial dilutions of the Phos-A peptide were tested as in Figure 3. The A-mut peptide was added at the concentration equivalent to that of Phos-A without dilution (10 µM). The phosphorylation reaction products were analyzed using an anti-phospho-Ser33/Ser37/Thr41 β-catenin antibody. *E*. Inhibition of GSK3 phosphorylation of β-catenin by Phos-A was independent of Axin. Four-fold serial dilutions of the Phos-A peptide (10 µM, 2.5 µM, and 0.63 µM) were included in the β-catenin phosphorylation assay in the absence or presence of Axin. The A-mut peptide was added at the concentration equivalent to that of Phos-A without dilution (10 µM). The phosphorylation reaction products were analyzed using an anti-phospho-Ser33/Ser37/Thr41 β-catenin antibody. Note that in order to achieve and visualize β-catenin phosphorylation by GSK3 in the absence of Axin (lanes 1–5), 5-fold excess amount of GSK3 (2.2 µM) was employed compared to that in the presence of Axin (lanes 6–10), and the film was overexposed. *F*. β-catenin Ser45 phosphorylation by CK1 was not affected by Phos-A, used at 10 µM and 2.5 µM. A-mut was at 10 µM. The phosphorylation reaction products were analyzed using an anti-phospho-Ser33/Ser37/Thr41 β-catenin antibody and an anti-phospho-Ser45 β-catenin antibody.

Since Axin is a scaffolding protein critical for GSK3 phosphorylation of β-catenin and binds to the phosphorylated PPPSPXS motif [34], [35], [37], we considered the possibility whether inhibition of β-catenin phosphorylation by the phosphorylated PPPSPXP motif involves the binding between Axin and the phosphorylated PPPSPXS motif. To this end we first employed an Axin mutant, AxinΔDIX (Figure 4A and 4B), which lacks the so-called DIX domain required for Axin-binding to LRP5/6 [36] and therefore does not associate with phosphorylated LRP6 or the PPPSPXP motif (H. H and X. H., unpublished results). β-catenin phosphorylation by GSK3 in vitro was promoted by AxinΔDIX as effectively as the wild type Axin; but unexpectedly, this reaction was inhibited by Phos-A in a similar dose-dependent manner in the presence of either the wild type Axin or AxinΔDIX (Figure 4C). This result implies that Phos-A inhibition of β-catenin phosphorylation by GSK3 may not involve its binding to Axin. To address this issue further, we tested another Axin fragment, Axin(351-701) (Figure 4A and 4B), which contains only the β-catenin- and GSK3-binding domains of Axin and promote β-catenin phosphorylation by GSK3 [42]. Indeed β-catenin phosphorylation by GSK3 was promoted by Axin(351-701) similarly to that by the wild type Axin, and importantly, was inhibited by Phos-A in a dose-dependent manner regardless of the presence of Axin or Axin(351-701) (Figure 4D). These results suggest that phosphorylated PPPSPXS peptide inhibited β-catenin phosphorylation by GSK3 via a manner that is independent of PPPSPXS-binding to Axin, perhaps by inhibiting GSK3 directly.

As demonstrated in Figure 1, β-catenin phosphorylation by GSK3 in vitro was greatly enhanced by the presence of Axin [9], [42]. However, GSK3 can nonetheless phosphorylate β-catenin in vitro, although less efficiently, in the absence of Axin [5], [9], [42]. Taking advantage of this property, we found that β-catenin phosphorylation at Ser33/Ser37/Thr41 by GSK3 in the absence of Axin was also inhibited by the Phos-A peptide in a dose-dependent manner (Figure 4E). As in the presence of Axin (Figure 3B), the A-mut peptide had little or no effect on β-catenin phosphorylation by GSK3 in the absence of Axin (Figure 4E). These results suggest that phosphorylated PPPSPXS motif directly inhibits GSK3 phosphorylation of β-catenin.

CK1 phosphorylates β-catenin at Ser45, which is the priming site for β-catenin Ser33/Ser37/Thr41 phosphorylation by GSK3 [5]. In addition, CK1 also phosphorylates LRP6 at the second Ser residue in the PPPSPXS motif [35], [38]. Therefore we tested whether the dually phosphorylated PPPSPXS peptide had any effect on β-catenin Ser45 phosphorylation by CK1. Although the Phos-A peptide inhibited β-catenin phosphorylation at Ser33/Ser37/Thr41 by GSK3, it had no effect on β-catenin phosphorylation at Ser45 by CK1 (Figure 4F). The A-mut peptide had little or no effect on β-catenin phosphorylation at Ser33/Ser37/Thr41 by GSK3 or at Ser45 by CK1 (Figure 4F). These results together suggest that the dually phosphorylated PPPSPXS motif can inhibit β-catenin phosphorylation at Ser33/Ser37/Thr41 by GSK3 independent of Axin, but it does not affect β-catenin phosphorylation at Ser45 by CK1.

### The dually phosphorylated PPPSPXS peptide inhibits the phosphorylation of glycogen synthase and Tau by GSK3

**Figure 5:**
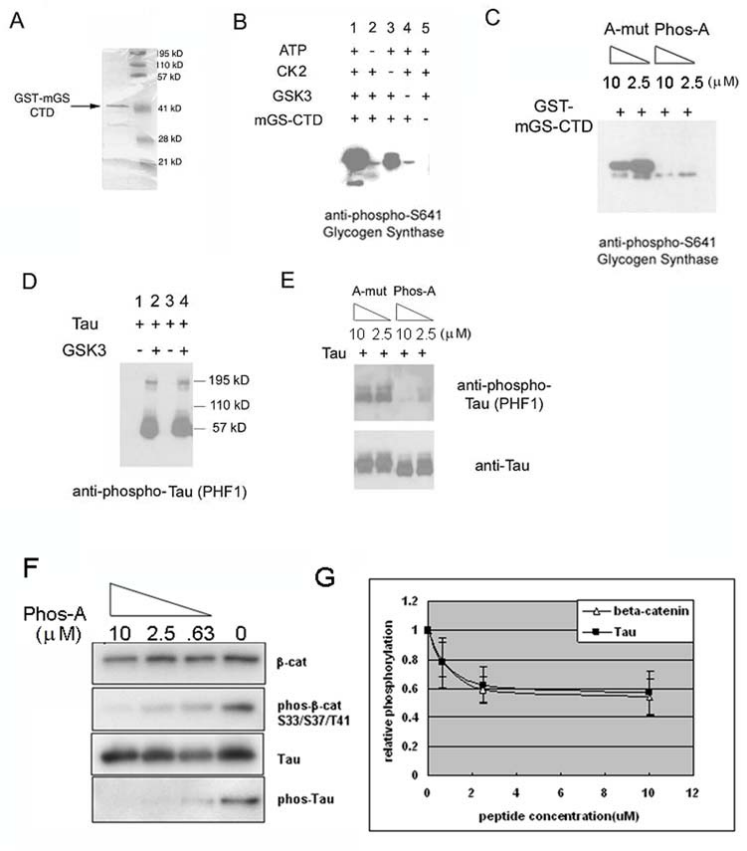
The phosphorylated PPPSPXS peptide inhibits phosphorylation of glycogen synthase and Tau by GSK3. *A*. Recombinant GST-tagged mouse glycogen synthase carboxyl-terminal domain (mGS-CTD) was expressed in bacteria, and purified by glutathione agarose resin. *B*. In vitro reconstitution of GS phosphorylation by CK2 and GSK3. The phosphorylation reaction products were analyzed using an anti-phospho-Ser641 GS antibody. *C*. GS phosphorylation at Ser641 was inhibited by Phos-A, but not by A-mut. The phosphorylation reaction products were analyzed using an anti-phospho-Ser641 GS antibody. *D*. In vitro reconstitution of Tau phosphorylation by GSK3. The phosphorylation reaction products were analyzed using an anti-phospho-Tau antibody (PHF1). *E*. Tau phosphorylation was inhibited by Phos-A, but not A-mut. The phosphorylation reaction products were analyzed using an anti-phospho-Tau antibody (PHF1). *F* and *G*. Different concentrations of Phos-A inhibited β-catenin and Tau phosphorylation by GSK3 in a similar manner. A four-fold dilution of Phos-A was employed. The graph represents the average of three independent experiments.

Since the dually phosphorylated PPPSPXS peptide directly inhibited β-catenin phosphorylation at Ser33/Ser37/Thr41 by GSK3 independent of Axin, we tested whether this inhibition was specific for β-catenin or was broad for other GSK3 substrates, such as glycogen synthase (GS) and the Tau protein [44]. We expressed the mouse glycogen synthase carboxyl terminal domain (mGS-CTD, amino acid 585–738) as a GST-fusion protein in *E. coli*, and purified mGS-CTD by GST column affinity chromatography (Figure 5A). We reconstituted in vitro phosphorylation for mGS-CTD by incubating it together with GSK3 and the priming casein kinase 2 (CK2) [44] in the presence of ATP and MgCl_2_. When CK2 and GSK3 were both present, mGS-CTD was strongly phosphorylated at Ser641 (Figure 5B, lanes 1 and 6). When CK2 was absent from the reaction, the phosphorylation of mGS-CTD at Ser641 by GSK3 was significantly reduced (Figure 5B, lane 3), recapitulating CK2 priming phosphorylation-dependent GS phosphorylation by GSK3. We found that the Phos-A peptide, but not the A-mutant peptide, inhibited mGS-CTD phosphorylation by GSK3 at Ser641 (Figure 5C). GSK3 also phosphorylated purified Tau protein in vitro, which does not require priming phosphorylation (Figure 5D), as detected by the anti-phospho-Tau antibody PHF1 (specific for Tau phosphorylated at Ser396 and Ser404) [45]. When the Phos-A peptide was added to the in vitro assay, Tau phosphorylation was greatly reduced, whereas the A-mut peptide had little effect (Figure 5E). In addition, when Phos-A peptide was titrated in the phosphorylation assay performed side-by-side for Tau and β-catenin (without Axin but with CK1 priming phosphorylation), indistinguishable dose-dependent inhibition of GSK3 by Phos-A was reproducibly observed (Figure 5F and 5G). These results suggest that the phosphorylated PPPSPXS motif inhibits GSK3 kinase activity towards multiple substrates.

### The dually phosphorylated PPPSPXS peptide can activate β-catenin signaling in Xenopus embryos

**Figure 6:**
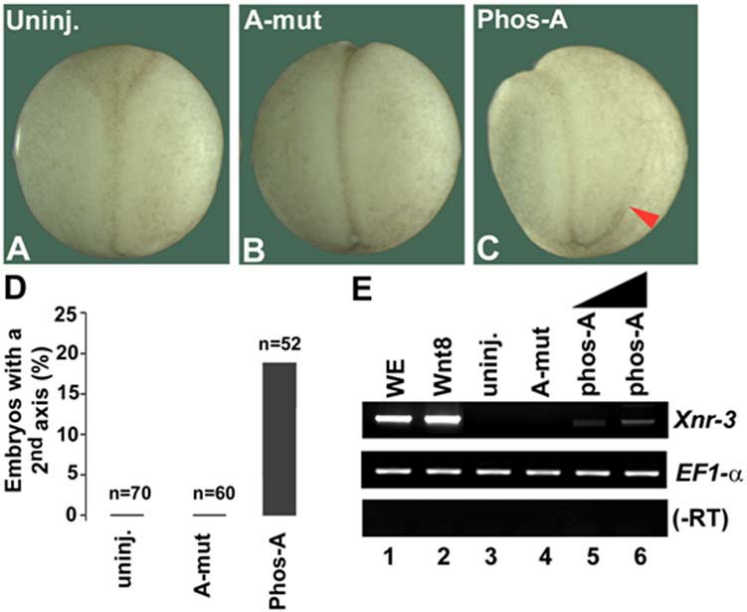
The Phos-A but not the A-mut peptide induces axis duplication and Xnr3 expression in Xenopus embryos. *A, B, C, D*. Uninjected embryo (A), A-mut-injected embryo (B), and Phos-A-injected embryo (C) shown at neural fold stage. The duplicated partial axis is labeled by the red arrowhead. Ventrally injected Phos-A (4.8 ng/embryo) induced axis duplication in 19% embryos (10 of 52). A-mut (4.8 ng/embryo) did not induce axis duplication (0 of 60). Three independent experiments were combined (D). *E*. Phos-A (3 and 4.8 ng/embryo) but not A-mut (4.8 ng/embryo) induced Xnr3 expression in animal pole explants, as assayed by RT-PCR. Xenopus Wnt8 RNA injection (8 pg/embryo) served as a positive control. The activity of the Phos-A peptide was significantly weaker than that of Wnt8 RNA, likely due to dilution, proteolysis and/or dephosphorylation in the embryo in the absence of any de novo synthesis. WE: whole embryo. EF1-a: loading control. –RT: without reverse transcriptase.

As the dually phosphorylated Phos-A peptide inhibits GSK3 phosphorylation of β-catenin in vitro, we examined whether Phos-A could inhibit GSK3 and activate β-catenin signaling in vivo. It has been well established that inhibition of GSK3, such as via a dominant-negative GSK3 mutant, activates Wnt/β-catenin signaling and induces axis duplication in Xenopus embryos [46], [47], [48]. We found that injection of the Phos-A peptide, but not the A-mut peptide, in Xenopus embryos induced partial axis duplication and the expression of Xnr3, a direct downstream target gene for β-catenin signaling [49] (Figure 6). Although the activity of the injected Phos-A peptide was significantly weaker compared to that of injected Xwnt8 mRNA (Figure 6), this was not unexpected since the phospho-peptide was likely subjected to dilution, proteolysis and/or dephosphorylation by proteases and/or phosphatases in the embryo in the absence of de novo synthesis. Importantly the A-mut peptide had no axis- or Xnr3-inducing activity in Xenopus embryos. These experiments demonstrate that the dually phosphorylated PPPSPXS peptide is able to activate Wnt/β-catenin signaling in embryos, presumably via inhibition of GSK3 phosphorylation of β-catenin in vivo.

## Discussion

Although it is known that the activated Wnt/Frizzled/LRP6 receptor complex results in inhibition of β-catenin degradation, the underlying molecular mechanism has remained unclear. One critical initiating event is Wnt-induced LRP6 phosphorylation at multiple PPPSPXS motifs [34], [35]. The phosphorylation of the PPPSPXS motif is sequentially carried out by GSK3 and CK1 [35], [38], and is under the control of the Frizzled receptor and its downstream partner Dishevelled protein [39], [40]. The dually phosphorylated PPPSPXS motif in turn provides a docking site for Axin [34], [35], thereby recruiting Axin and likely associated proteins to the Frizzled-LRP6 receptor complex [36]. But how these events lead to inhibition of β-catenin phosphorylation by the Axin-GSK3 complex has been poorly understood.

We attempted to address this question via a β-catenin phosphorylation assay reconstituted in vitro using recombinant β-catenin, GSK3, CK1, and Axin. This in vitro system recapitulated key features of β-catenin phosphorylation in vivo, such that GSK3 phosphorylation of β-catenin at Ser33/Ser37/Thr41 is greatly enhanced by the presence of Axin and by priming phosphoryaltion of β-catenin at Ser45 by CK1 (Figure 1). Given that a single PPPSPXS motif upon phosphorylation is sufficient to activate β-catenin signaling in both mammalian cells and Xenopus embryos [34], [35], [37], we tested whether phosphorylated PPPSPXS motifs from LRP6 had any effect on β-catenin phosphorylation in this in vitro assay. We found that each of four dually phosphorylated PPPSPXS peptides we examined, motifs A, C, D and E (Figure 2), exhibits inhibition of β-catenin phosphorylation at Ser33/Ser37/Thr41 by GSK3 in a dose-dependent manner (Figure 3). We speculate that motif B may exhibit the same property, although we have not synthesized and tested a motif B peptide in our assay. Several aspects of this inhibition appear to correlate well with the properties of Wnt/LRP6 regulation of β-catenin phosphorylation in vivo: (i) a corresponding mutant peptide, PPPAPXA (Figure 2), has little, if any, inhibitory effect on β-catenin phosphorylation by GSK3 under the identical experimental condition (Figure 3). This correlates well with the fact that the dually phosphorylated PPPSPXS motif upon transferred to a heterologous receptor is sufficient to activate β-catenin signaling in vivo, whereas the PPPAPXA mutant is completely inactive [34], [35], [37]. In addition, other control peptides including HA and 14-3-3BP (a dually phosphorylated peptide) do not have any inhibitory activity in the β-catenin phosphorylation assay (Figure 3); (ii) the ability of dually phosphorylated PPPSPXS peptides to inhibit β-catenin phosphorylation by GSK3 correlates well with their differential ability to activate β-catenin signaling tested in vivo [37]. Thus the Phos-D peptide, which has an atypical CPPSPXS motif (Figure 2), has the weakest ability to inhibit β-catenin phosphorylation by GSK3 in vitro (Figure 3) and also the least ability to activate β-catenin signaling in vivo when it is transferred to a heterologous receptor [37]. Further, this result is also in line with that from scanning mutagenesis of the PPPSPXS motif, which has demonstrated a critical role of the first proline in the PPPSPXS motif such that its replacement by alanine or cysteine drastically diminished the signaling activity of the motif in vivo [37]; (iii) the dually phosphorylated PPPSPXS motif inhibits β-catenin phosphorylation at Ser33/Ser37/Thr41 by GSK3 but not Ser45 phosphorylation by CK1 in vitro (Figure 4). This correlates with the fact that Wnt signaling primarily inhibits β-catenin phosphorylation at Ser33/Ser37/Thr41 by GSK3 but not Ser45 phosphorylation by CK1 [5], [50]; (iv) finally we found that injection of a phosphorylated PPPSPXS peptide, but not the mutant PPPAPXA peptide, into Xenopus embryos is able to induce axis duplication and the expression of Xnr3, a Wnt/β-catenin target gene (Figure 6), consistent with the notion that phosphorylated PPPSPXS inhibits GSK3 in vivo. These results together suggest that our in vitro observations with the PPPSPXS peptides likely reflect, at least in part, the function of these motifs in activated LRP6 in vivo.

Although Axin is required for β-catenin phosphorylation by GSK3 both in vivo and in vitro (Figure 1), and Axin interacts with phosphorylated PPPSPXS motifs [34], [35], we were surprised to find that inhibition of GSK3 phosphorylation of β-catenin by the phoshorylated PPPSPXS peptide is independent of Axin function (Figure 4). Two different Axin mutants, AxinΔDIX and Axin(351-701), are each fully capable of promoting β-catenin phosphorylation by GSK3, and this phosphorylation is inhibited by the phosphorylated PPPSPXS peptide to the extent similar to that in the presence of the wild type Axin (Figure 4). Neither of these Axin mutants is expected to bind to the phosphorylated PPPSPXS motif because the DIX domain, which is deleted in both Axin mutants (Figure 4), is required for such binding [36; H. H. and X. H., unpublished results]. This point was best illustrated by Axin(351-701), which harbors only the GSK3- and β-catenin-binding domains of Axin (Figure 4). These results led us to suspect that GSK3 is directly inhibited by the dually phosphorylated PPPSPXS peptide. Indeed, when a higher concentration of GSK3 is applied in vitro together with CK1 for priming phosphorylation, β-catenin is phosphorylated at Ser33/Ser37/Thr41 (albeit less effectively) in the absence of Axin as previously reported [5], and this β-catenin phosphorylation by GSK3 is inhibited by the phosphorylated PPPSPXS peptide (Figure 4), indicating a direct inhibition of GSK3 without the involvement of Axin.

Cselenyi *et al* recently reported that the recombinant LRP6 intracellular domain directly inhibits GSK3 phosphorylation of β-catenin in Xenopus egg extracts and in a reconstituted in vitro kinase assay similar to the one employed in this study [41]. They found that the inhibition of GSK3 by the LRP6 intracellular domain is direct and Axin-independent, and requires the intact PPPSPXS motifs [41]. While Cselenyi *et al* tested the LRP6 intracellular domain at a single concentration, our experiments examined each individual phosphorylated PPPSPXS motif, which represents the minimal signaling module as our previous studies have suggested [34], [35], [37], and we demonstrated dose-dependent inhibition of GSK3 by these phospho-PPPSPXS peptides at multiple concentrations. Overall our results are in good agreement with the main conclusions by Cselenyi *et al*. However, one noticeable difference exists between the results by Cselenyi *et al* and ours regarding the inherent (or the lack of) specificity of phospho-LRP6 inhibition of GSK3 phosphorylation. Cselenyi *et al* reported that the LRP6 intracellular domain specifically inhibits GSK3 phosphorylation of β-catenin, but not of Tau [41]. By contrast, we found that the phosphorylated PPPSPXS peptide also inhibits GSK3 phosphorylation of Tau and glycogen synthase (GS, with priming phosphorylation by CK2), and indeed GSK3 phosphorylation of β-catenin and Tau appears to be similarly inhibited by different concentrations of the phospho-PPPSPXS peptide (Figure 5). Our results suggest a general inhibition of GSK3 activity by the phosphorylated PPPSPXS peptide, and are consistent with an earlier study demonstrating that the LRP6 intracellular domain can inhibit GSK3 phosphorylation of both β-catenin and Tau [51]. We note that our findings are fully compatible with specific inhibition of β-catenin phosphorylation by Wnt signaling (see below).

One potential mechanism for the observed inhibition of GSK3 by the phosphorylated PPPSPXS peptide is substrate competition, given that the PPPSPXS motif is a substrate for GSK3 [35], [51], and may compete with β-catenin for GSK3. However this mechanism does not easily explain why the phosphorylated PPPSPXS peptide has no inhibitory effect on β-catenin phosphorylation at Ser45 by CK1, considering the fact that PPPSPXS is also a substrate for CK1 [35], [38]. Another potential mechanism, which is not mutually exclusive with the substrate competition model, is that the phosphorylated PPPSPXS motif may directly bind to GSK3 (but not CK1) and inhibit GSK3 kinase activity. Indeed GSK3 has been identified as an LRP6-binding protein [35] and vice versa [51] from yeast two-hybrid screens and can be co-precipitated with LRP6 [35], [51], and GSK3 associated with LRP6 exhibits reduced kinase activity [51]. We note that each LRP6 molecule has five phosphorylated PPPSPXS motifs and that LRP6 upon Wnt activation may multimerize [40]. Therefore a high local concentration of phosphorylated PPPSPXS motifs likely exists and their proximity to GSK3 via LRP6-Axin association may provide significant binding and inhibition to GSK3 in vivo even if the interaction between each phosphorylated PPPSPXS motif and GSK3 may not be particularly strong. We also note that the molar concentration of phospho-PPPSPXS peptides used in our GSK3 inhibition assays in vitro were at 0.4-, 1.5-, 6-, and 24-fold of that of GSK3, and that interestingly the phospho-peptides at 6-fold concentration relative to that of GSK3 exhibits significant inhibition towards GSK3, correlating remarkably with the five PPPSPXS motifs in each LRP6 protein. Therefore it seems that the relative concentrations of phospho-PPPSPXS peptides and GSK3 in our in vitro assays may be within a range conceivable in vivo.

**Figure 7:**
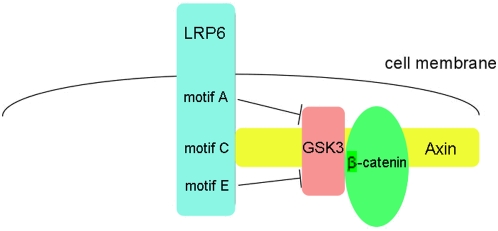
A working model for LRP6 inhibition of β-catenin phosphorylation by the Axin-GSK3 complex. While one of the five phosphorylated PPPSPXS motifs of LRP6 physically interacts with Axin, other phosphorylated PPPSPXS motifs may directly inhibit GSK3 phosphorylation of β-catenin in the Axin complex. Axin-binding to motif C is drawn arbitrarily. See Discussion for details.

In summary, we propose a working model to link LRP6 activation and the inhibition of β-catenin phosphorylation. When activated by Wnt, PPPSPXS motifs in LRP6 intracellular domain are phosphorylated by GSK3 and CK1, and they act together to recruit Axin-GSK3 into the Wnt receptor complex. When Axin is bound to one of the phosphorylated PPPSPXS motifs, other phosphorylated PPPSPXS motifs in the vicinity of the Axin-GSK3 complex directly inhibit GSK3 phosphorylation of β-catenin (Figure 7). Several considerations suggest the feasibility of this model. First, although each phosphorylated PPPSPXS motif can bind to Axin [34], [37], it is unlikely that these five PPPSPXS motifs clustered within a 120-residue region of LRP6 intracellular domain bind simultaneously to five molecules of Axin, which has 863 amino acids. Rather, these five PPPSPXS motifs together may provide a high local concentration of docking sites for a single Axin molecule, thereby ensuring a tight association between LRP6 and Axin. In this scenario multiple phosphorylated PPPSPXS motifs in each LRP6 are available for direct inhibition of GSK3. This model implies that the five PPPSPXS motifs when fully phosphorylated may have two major functions, i.e., to ensure the binding of Axin to LRP6 and to carry out inhibition of GSK3 phosphorylation of β-catenin. This notion is consistent with the finding by others and us that the coorperativity of the five PPPSPXS motifs are critical for LRP6 signaling function [37], [43]. Secondly, this model explains LRP6 signaling specificity in the inhibition of β-catenin phosphorylation, since GSK3 phosphorylation of other substrates such as GS and Tau occurs outside the Axin complex and is not in the proximity to LRP6 upon Wnt stimulation, and therefore will not be affected by the phosphorylated PPPSPXS motif under the physiological condition. Thirdly, direct inhibition of GSK3 by phospho-PPPSPXS motifs within LRP6 is consistent with a recent observation that upon Wnt stimulation dephosphorylated β-catenin is observed at the plasma membrane together with the activated LRP6 [52].

We note that our above model (Figure 7) does not exclude the possibility that there also exist other mechanisms by which activated Wnt-Frizzled-LRP6 signaling leads to the inhibition of β-catenin phosphorylation by GSK3. For example, dissociation of GSK3 from the Axin complex upon Wnt signaling has been suggested to be one such mechanism [53]. These parallel mechanisms may operate together to ensure prevention of GSK3 phosphorylation of β-catenin upon Wnt stimulation. This may be analogous to parallel mechanisms that promote GSK3 phosphorylation of β-catenin in the absence of Wnt stimulation, such as by GSK3 and CK1 phosphorylation of Axin and APC [54].

While this manuscript was being revised for publication Piao et al reported that a dually phosphorylated PPPSPXS peptide (essentially identical to Phos-A in our study) inhibits GSK3 phosphorylation of both β-catenin and Axin in vitro, and a LRP6 intracellular fragment containing a single PPPSPXS motif inhibits GS phosphorylation when overexpressed in the cytoplasm [55]. Consistent with our findings, Piao et al propose that phosphorylated PPPSPXS motif is a general inhibitor of GSK3 activity.

## Materials and Methods

### Expression plasmids and baculoviruses

The pGEX4T1 vector expressing wild-type β-catenin (GST-tagged) was described [17]. The mouse glycogen synthase C-terminal domain (mGS-CTD, amino acid 585–738) was cloned into the pGEX4T1 vector (GE Healthcare) using standard cloning procedures. pCS2+Axin, pCS2+AxinΔDIX, and pCS2+Axin(351-701) (all were Flag-tagged) were cloned using standard cloning procedures. The baculovirus expressing the Flag-tagged CK1 was a kind gift from Wade Harper (Harvard Medical School). Baculoviruses expressing 6xHis-tagged GSK3 and MBP-tagged Axin were kind gifts from Ethan Lee (Vanderbilt University).

### Protein expression and purification

The GST-tagged β-catenin and GST-tagged mGS-CTD were expressed by transforming expression plasmids into BL21 (DE3) cells, grown in LB Broth, and induced by 0.5 mM IPTG. Bacterial cells were then lysed by three cycles of freeze-thaw. The cell lysate was centrifuged at 14,000 rpm for 30 min, and the supernatant was incubated with glutathione agarose resin (Sigma). The bound proteins were washed by buffer and eluted by 7 mM glutathione. Baculoviruses for Flag-tagged CK1, His-tagged GSK3, and MBP-tagged Axin were amplified in the Sf9 insect cells (Invitrogen), and then used to infect the High-Five insect cells (Invitrogen), which were harvested two days after infection and lysed by freeze-thaw cycles. The M2 anti-Flag agarose resin (Sigma) was used to purify Flag-tagged CK1. The amylose resin (New England Biolab) was used to purify MBP-tagged Axin. The Ni-NTA agarose resin was used to purify His-tagged GSK3. The purified Tau protein was purchased from Calbiochem. Flag-Axin, Flag-AxinΔDIX, and Flag-Axin(351-701) were overexpressed by transfecting HEK293T cells with the respective expression plasmids, and purified using M2 anti-Flag agarose resin (Sigma) from cell extracts lysed in buffer with 0.5% NP40.

### Peptide synthesis

The HA, 14-3-3BP, Phos-A, A-mut, Phos-C, Phos-D, and Phos-E peptides were synthesized by Tufts University Core Facility, purified by reverse phase HPLC, and their identities were confirmed by mass spectrometry. The synthesized peptides were dissolved in Tris buffer (pH 8.0) to 500 µM, and dialyzed by the 3,500 Dalton Molecular-Weight-Cut-Off Slide-A-Lyzer MINI dialysis units overnight to remove chemicals used in peptide synthesis. Recovery of peptides after dialysis was estimated to be 30% for all peptides using dot-blotting analysis via respective antibodies [34], [35], [37], with undialyzed peptides as standards. Phos-A and 14-3-3BP peptides were synthetically dually phosphorylated. Phos-C, Phos-D, and Phos-E peptides were synthesized as singly phosphorylated at the GSK3 site (PPPSPXS), and were further phosphorylated by CK1 at the second serine site (PPPSPXS) at 37°C for 3 hours in vitro.

### Mammalian cell transfection

Transfections were done in HEK293T cells in 6-well plates using Fugene-6 (Roche). 48 hours after transfection, cells were lysed in a buffer containing 10 mM Tris (pH 8.0), 150 mM NaCl, 5 mM EDTA, 10 mM NaF, and 0.5% NP-40 with a cocktail of protease inhibitors. The cell lysates were centrifuged at 14,000 rpm for 10 minutes, and the supernatant of the cell lysates were taken for further studies.

### In vitro phosphorylation assay

The phosphorylation assays were performed in buffer containing: 25 mM Hepes pH 7.5, 20 mM β-glycerophosphate, 25 mM MgCl_2_, 2 mM DTT, and 10 mM ATP (pH 7.0), in a total volume of 15 µl at 37°C for 3 hours. For Axin-dependent phosphorylation of β-catenin, 0.43 µM of GSK3, 0.54 µM of CK1α, 0.21 µM of Axin, and 0.73 µM of β-catenin were used in each assay. For Axin-independent phosphorylation of β-catenin, the condition was the same except that 2.2 µM of GSK3 (and no Axin) were used in each assay. For phospho-peptide (or control peptide) inhibition experiments, 1 µl of the dialyzed peptide (final concentration: 10 µM) or four-fold serially diluted peptide (final concentrations: 2.5 µM, 0.63 µM, and 0.16 µM) was used in each assay. For GS phosphorylation, 0.50 µM of CK2 proteins, 0.43 µM of GSK3, and 0.8 µM of GST-mGS-CTD were used in each reaction. For Tau phosphorylation, 0.43 µM of GSK3 and 0.8 µM of Tau protein were used in each reaction. Each in vitro assay was repeated three or more times.

### Antibodies and western blotting

The supernatant of cell lysates or kinase reaction products were examined by SDS-PAGE, and analyzed by Western blotting using Immobilon-P membrane (Millipore). The membranes were incubated in blocking buffer (5% nonfat dry milk in TBS buffer with 0.1% Tween-20) for 1 hour at room temperature, and then were incubated with primary antibodies diluted in 1% BSA in the TBS/Tween-20 buffer for 1 hour, followed by incubation with horseradish peroxide-conjugated secondary antibodies diluted at 1∶10,000 in 1% BSA in the TBS/Tween-20 buffer for 30 minutes. Protein detection was performed using the ECL system (Amersham Pharmacia). The following antibodies were used (values in parentheses are the dilution ratios used for Western blotting): anti-phospho-Ser33/Ser37/Thr41 β-catenin (1∶1000) from Cell Signaling (9561S); anti-phospho-Ser45 β-catenin (1∶1000) from Cell Signaling (9564S); anti-β-catenin (1∶1000) from BD Biosciences (610153); anti-phospho-Ser641 glycogen synthase (1∶1000) from Cell Signaling (3891S); and anti-Tau (1∶1000) from Cell Signaling (4019). The PHF1 anti-phosphor-Tau antibody was a kind gift from Peter Davies (Albert Einstein School of Medicine).

### Xenopus embryos

Embryo manipulations and RT-PCR were described previously [30]. Embryos were injected with the Phos-A or A-mutant peptide in the ventral marginal region at 8-cell stage for the axis-duplication assay, or in the animal region at 4-cell stage for the animal pole explant assay.

## References

[1] Logan C, Nusse R (2004). The Wnt signaling pathway in development and disease Annu.. Rev Cell Dev Biol.

[2] Moon RT, Bowerman B, Boutros M, Perrimon N (2002). The promise and perils of Wnt signaling through β-catenin.. Science.

[3] van Es JH, Barker N, Clevers H (2003). You Wnt some, you lose some: oncogenes in the Wnt signaling pathway.. Curr Opin Genet Dev.

[4] Polakis P (2000). Wnt signaling and cancer.. Genes Dev.

[5] Liu C, Li Y, Semenov M, Han C, Baeg GH (2002). Control of β-catenin phosphorylation/degradation by a dual-kinase mechanism.. Cell.

[6] Zeng L, Fagotto F, Zhang T, Hsu W, Vasicek TJ (1997). The mouse Fused locus encodes Axin, an inhibitor of the Wnt signaling pathway that regulates embryonic axis formation.. Cell.

[7] Behrens J, Jerchow BA, Wurtele M, Grimm J, Asbrand C (1998). Functional interaction of an axin homolog, conductin, with β-catenin, APC, and GSK3β.. Science.

[8] Hart MJ, de los Santos R, Albert IN, Rubinfeld B, Polakis P (1998). Downregulation of β-catenin by human Axin and its association with the APC tumor suppressor, β-catenin and GSK3 β.. Curr Biol.

[9] Ikeda S, Kishida S, Yamamoto H, Murai H, Koyama S (1998). Axin, a negative regulator of the Wnt signaling pathway, forms a complex with GSK-3 and β-catenin and promotes GSK-3-dependent phosphorylation of β-catenin.. EMBO J.

[10] Sakanaka C, Weiss JB, Williams LT (1998). Bridging of β-catenin and glycogen synthase kinase-3β by axin and inhibition of β-catenin-mediated transcription.. Proc Natl Acad Sci USA.

[11] Itoh K, Krupnik VE, Sokol SY (1998). Axis determination in Xenopus involves biochemical interactions of axin, glycogen synthase kinase 3 and β-catenin.. Curr Biol.

[12] Salic A, Lee E, Mayer L, Kirschner MW (2000). Control of β-catenin stability: reconstitution of the cytoplasmic steps of the wnt pathway in Xenopus egg extracts.. Mol Cell.

[13] Amit S, Hatzubai A, Birman Y, Andersen JS, Ben-Shushan E (2002). Axin-mediated CKI phosphorylation of β-catenin at Ser 45: a molecular switch for the Wnt pathway.. Genes Dev.

[14] Yanagawa S, Matsuda Y, Lee JS, Matsubayashi H, Sese S (2002). Casein kinase I phosphorylates the Armadillo protein and induces its degradation in Drosophila.. EMBO J.

[15] Wu G, He X (2006). Threonine 41 in β-catenin serves as a key phosphorylation relay residue in β-catenin degradation.. Biochemistry.

[16] Winston JT, Strack P, Beer-Romero P, Chu CY, Elledge SJ (1999). The SCFβ-TRCP-ubiquitin ligase complex associates specifically with phosphorylated destruction motifs in IkappaBalpha and β-catenin and stimulates IkappaBalpha ubiquitination in vitro.. Genes Dev.

[17] Liu C, Kato Y, Zhang Z, Do VM, Yankner BA (1999). β-Trcp couples β-catenin phosphorylation-degradation and regulates Xenopus axis formation.. Proc Natl Acad Sci USA.

[18] Hart M, Concordet JP, Lassot I, Albert I, del los Santos R (1999). The F-box protein β-TrCP associates with phosphorylated β-catenin and regulates its activity in the cell.. Curr Biol.

[19] Kitagawa M, Hatakeyama S, Shirane M, Matsumoto M, Ishida N (1999). An F-box protein, FWD1, mediates ubiquitin-dependent proteolysis of β-catenin.. EMBO J.

[20] Latres E, Chiaur DS, Pagano M (1999). The human F box protein β-Trcp associates with the Cul1/Skp1 complex and regulates the stability of β-catenin.. Oncogene.

[21] Wu G, Xu G, Schulman BA, Jeffrey PD, Harper JW (2003). Structure of a β-TrCP1-Skp1-β-catenin complex: destruction motif binding and lysine specificity of the SCF(β-TrCP1) ubiquitin ligase.. Mol Cell.

[22] Wu G, Liu C, He X (2004). Ozz; a new name on the long list of β-catenin's nemeses.. Mol Cell.

[23] Aberle H, Bauer A, Stappert J, Kispert A, Kemler R (1997). β-catenin is a target for the ubiquitin-proteasome pathway.. EMBO J.

[24] Orford K, Crockett C, Jensen JP, Weissman AM, Byers SW (1997). Serine phosphorylation-regulated ubiquitination and degradation of β-catenin.. J Biol Chem.

[25] Behrens J, von Kries JP, Kuhl M, Bruhn L, Wedlich D (1996). Functional interaction of β-catenin with the transcription factor LEF-1.. Nature.

[26] Huber O, Korn R, McLaughlin J, Ohsugi M, Herrmann BG (1996). Nuclear localization of β-catenin by interaction with transcription factor LEF-1.. Mech Dev.

[27] Molenaar M, van der Wetering M, Oosterwegel M, Peterson-Maduro J, Godsave S (1996). XTcf-3 transcription factor mediates β-catenin-induced axis formation in Xenopus embryos.. Cell.

[28] He TC, Sparks AB, Rago C, Hermeking H, Zawel L (1998). Identification of c-MYC as a target of the APC pathway.. Science.

[29] Tetsu O, McCormick F (1999). Β-catenin regulates expression of cyclin D1 in colon carcinoma cells.. Nature.

[30] Tamai K, Semenov M, Kato Y, Spokony R, Liu C (2000). LDL-receptor-related proteins in Wnt signal transduction.. Nature.

[31] Wehrli M, Dougan ST, Caldwell K, O'Keefe L, Schwartz S (2000). arrow encodes an LDL-receptor-related protein essential for Wingless signalling.. Nature.

[32] Pinson KI, Brennan J, Monkley S, Avery BJ, Skarnes WC (2000). An LDL-receptor-related protein mediates Wnt signalling in mice.. Nature.

[33] He X, Semenov M, Tamai K, Zeng X (2004). LDL receptor-related proteins 5 and 6 in Wnt/β-catenin signaling: arrows point the way.. Development.

[34] Tamai K, Zeng X, Liu C, Zhang X, Harada Y (2004). A mechanism for Wnt coreceptor activation.. Mol Cell.

[35] Zeng X, Tamai K, Doble B, Li S, Huang H (2005). A dual-kinase mechanism for Wnt co-receptor phosphorylation and activation.. Nature.

[36] Mao J, Wang J, Liu B, Pan W, Farr G (2001). Low-density lipoprotein receptor-related protein-5 binds to Axin and regulates the canonical Wnt signaling pathway.. Mol Cell.

[37] MacDonald B, Yokota C, Tamai K, Zeng X, He X (2008). Wnt signal amplification: activity, cooperativity and regulation of multiple intracellular PPPSP motifs in the Wnt coreceptor LRP6.. J Biol Chem.

[38] Davidson G, Wu W, Shen J, Bilic J, Fenger U (2005). Casein kinase 1 gamma couples Wnt receptor activation to cytoplasmic signal transduction.. Nature.

[39] Zeng X, Huang H, Tamai K, Zhang X, Harada Y (2008). Initiation of Wnt signaling: control of Wnt coreceptor Lrp6 phosphorylation/activation via frizzled, dishevelled and axin functions.. Development.

[40] Bilic J, Huang Y, Davidson G, Zimmermann T, Cruciat C (2007). Wnt induces LRP6 signalosomes and promotes dishevelled-dependent LRP6 phosphorylation.. Science.

[41] Cselenyi C, Jernigan K, Tahinci E, Thorne C, Lee L (2008). LRP6 transduces a canonical Wnt signal independently of Axin degradation by inhibiting GSK3's phosphorylation of β-catenin.. Proc Natl Acad Sci USA.

[42] Dajani R, Fraser E, Roe S, Yeo M, Good V (2003). Structural basis for recruitment of glycogen synthase kinase 3β to the axin-APC scaffold complex.. EMBO J.

[43] Wolf J, Palmby T, Gavard J, Williams B, Gutkind S (2008). Multiple PPPS/TP motifs act in a combinatorial fashion to transduce Wnt signaling through LRP6.. FEBS Lett.

[44] Doble BW, Woodgett JR (2003). GSK-3: tricks of the trade for a multi-tasking kinase.. J Cell Sci.

[45] Greenberg SG, Davies P, Schein JD, Binder LI (1992). Hydrofluoric acid-treated tau PHF proteins display the same biochemical properties as normal tau.. J Biol Chem.

[46] He X, Saint-Jeannet JP, Woodgett JR, Varmus HE, Dawid IB (1995). Glycogen synthase kinase-3 and dorsoventral patterning in Xenopus embryos.. Nature.

[47] Dominguez I, Itoh K, Sokol SY (1995). Role of glycogen synthase kinase 3 beta as a negative regulator of dorsoventral axis formation in Xenopus embryos.. Proc Natl Acad Sci USA.

[48] Pierce SB, Kimelman D (1995). Regulation of Spemann organizer formation by the intracellular kinase Xgsk-3.. Development.

[49] Harland R, Gerhart J (1997). Formation and function of Spemann's organizer.. Annu Rev Cell Dev Biol.

[50] Doble B, Patel S, Wood G, Kockeritz L, Woodgett J (2007). Functional redundancy of GSK-3α and GSK-3β in Wnt/β-catenin signaling shown by using an allelic series of embryonic stem cell lines.. Dev Cell.

[51] Mi K, Dolan P, Johnson G (2006). The low density lipoprotein receptor-related protein 6 interacts with glycogen synthase kinase 3 and attenuates activity.. J Biol Chem.

[52] Hendriksen J, Jansen M, Brown CM, Velde H, Ham M (2008). Plasma membrane recruitment of dephosphorylated β-catenin upon activation of the Wnt pathway.. J of Cell Sci.

[53] Liu X, Rubin J, Kimmel A (2005). Rapid, Wnt-induced changes in GSK3β associations that regulate β-catenin stabilization are mediated by Galpha proteins.. Curr Biol.

[54] Kimelman D, Xu W (2006). β-catenin destruction complex: insights and questions from a structural perspective.. Oncogene.

[55] Piao S, Lee S, Kim H, Yum S, Stamos JL (2008). Direct inhibition of GSK3β by the phosphorylated cytoplasmic domain of LRP6 in Wnt/β-catenin signaling.. PLoS ONE.

